# Poly(vinylphosphonic acid‐*co*‐acrylic acid) hydrogels: The effect of copolymer composition on osteoblast adhesion and proliferation

**DOI:** 10.1002/jbm.a.36234

**Published:** 2017-10-24

**Authors:** Rebecca E. Dey, Ian Wimpenny, Julie E. Gough, David C. Watts, Peter M. Budd

**Affiliations:** ^1^ School of Chemistry The University of Manchester Manchester M13 9PL United Kingdom; ^2^ School of Materials The University of Manchester Manchester M13 9PL United Kingdom; ^3^ School of Medical Sciences and Photon Science Institute The University of Manchester Manchester M13 9PL United Kingdom

**Keywords:** vinylphosphonic acid, acrylic acid, hydrogel, bone tissue engineering, adhesion, proliferation, mechanical properties

## Abstract

There is a clinical need for a synthetic bone graft substitute that can be used at sites of surgical intervention to promote bone regeneration. Poly(vinylphosphonic acid‐*co*‐acrylic acid) (PVPA‐*co*‐AA) has recently been identified as a potential candidate for use in bone tissue scaffolds. It is hypothesized that PVPA‐*co*‐AA can bind to divalent calcium ions on bone mineral surfaces to control matrix mineralization and promote bone formation. In this study, hydrogels of PVPA‐*co*‐AA have been produced and the effect of copolymer composition on the structure and properties of the gels was investigated. It was found that an increase in VPA content led to the production of hydrogels with high porosities and greater swelling capacities. Consequently, improved cell adhesion and proliferation was observed on these hydrogels, as well as superior cell spreading morphologies. Furthermore, whereas poly(acrylic acid) gels were shown to be relatively brittle, an increase in VPA content created more flexible hydrogels that can be more easily molded into bone defect sites. Therefore, this work demonstrates that the mechanical and cell adhesion properties of PVPA‐*co*‐AA hydrogels can be tuned for the specific application by altering the copolymer composition. © 2017 The Authors Journal of Biomedical Materials Research Part A Published by Wiley Periodicals, Inc. J Biomed Mater Res Part A: 106A: 255–264, 2018.

## INTRODUCTION

Osteoporosis is a debilitating disease, characterized by porous, brittle bones and an increased risk of fractures. It is reported that osteoporosis results in >8.9 million fractures annually worldwide.^1^ The repair of large, nonunion bone defects can be very challenging in osteoporotic patients and a bone graft is often required. Most commonly, autografts and allografts are used.^2^ However, these techniques present many complications, including limitation of supply^3^ and rejection by the immune system.^2^ Therefore, there is a need for a synthetic bone graft substitute that can be used at sites of surgical intervention to promote bone regeneration.

Tissue engineering is a promising strategy that seeks to meet this need by producing bioactive scaffolds that can act as a temporary structural support as well as initiating a regenerative response. When designing synthetic scaffolds for bone tissue engineering, there are certain criteria that must be upheld. For example, scaffolds should be biocompatible and biodegradable with nontoxic breakdown products.^4^, ^5^ Scaffolds should also be highly porous to allow the infiltration of cells and nutrients,^6^, ^7^ while maintaining their mechanical integrity to withstand the forces exerted on them by the body.^8^ The ideal tissue scaffold would possess the osteoconductive and osteoinductive properties of the native extracellular matrix (ECM) of bone.^9^ The ECM is primarily composed of collagen fibers interspersed with hydroxyapatite (HA) crystals. It is believed that scaffolds which mimic the morphology of the ECM will produce a greater cellular response.^10^

Synthetic polymeric hydrogels, such as poly(ethylene glycol) (PEG)^11^, ^12^ and poly(vinyl alcohol) (PVA),^13^ have been studied extensively for use in biomedical applications, including drug delivery, wound healing, and tissue regeneration. Hydrogels can be defined as crosslinked polymeric networks with high water contents. The swelling of hydrogels in aqueous media is influenced by several factors, including hydrophilicity, chemical composition,^14^ and crosslink density.^15^ Although hydrogels suffer from low mechanical strength, their structure and elasticity allows the physical shape of hydrogels to be manipulated to fit any particular application site, making them ideal for use as bone graft substitutes.

Hydrogels which incorporate anionic polyelectrolytes, such as poly(acrylic acid) (PAA),^14^, ^16^ have gained significant attention for use in drug delivery systems due to the fact that their swelling behavior depends strongly on external stimuli,^17^, ^18^ such as pH, ionic strength, and temperature. At physiological pH, the acidic groups are negatively charged and so electrostatic repulsions occur within the polymer network, which leads to a significant degree of swelling. This allows for increased cell infiltration as well as diffusion and transport of nutrients within the gels.

Poly(vinylphosphonic acid) (PVPA) is another example of a negatively charged polyelectrolyte, which has emerged in recent years as a potential candidate for use in bone tissue scaffolds.^19^, ^20^, ^21^ It is hypothesized that PVPA acts as a mimic of bisphosphonates (BPs); a class of drugs used in the treatment of osteoporosis. The structure of BPs allows them to bind avidly to divalent calcium ions on the bone mineral surface, potentially enhancing bone mineralization.^22^ BPs also induce osteoclast apoptosis, leading to a net increase in bone formation.^23^ Bassi et al.^24^, ^25^ have produced electrospun scaffolds composed of PCL, functionalized with poly(vinylphosphonic acid‐*co*‐acrylic acid) (PVPA‐*co*‐AA). These scaffolds demonstrate an increase in surface wettability, matrix mineralization, and bone formation, when compared with PCL alone.

Although it has been shown that PVPA‐*co*‐AA leads to an increase in bone formation, the influence of copolymer composition is still unclear. In recent work by the authors, calcium chelation was investigated as a function of VPA content in the copolymer.^26^ It was found that PVPA‐*co*‐AA, with a VPA content of 30 mol %, led to the highest calcium binding. This suggests that this copolymer composition will have the most positive effect on matrix mineralization as well as significantly enhancing osteoblast adhesion and proliferation. Furthermore, Tan et al.^27^ have produced hydrogels of poly(vinylphosphonic acid‐*co*‐acrylamide) (PVPA‐*co*‐Am). These gels show an increase in swelling capabilities with increasing VPA content. The presence of VPA also had a substantial influence on the ability of the gels to attract proteins from cell culture medium.

In the present study, hydrogels of PVPA‐*co*‐AA were produced and investigated for their ability to promote adhesion and proliferation of an osteoblast‐like SaOS‐2 cell line. The effect of VPA content on the structural and mechanical properties of the hydrogels was also investigated.

## MATERIALS AND METHODS

### Materials

All chemicals were used without further purification unless otherwise stated. Vinylphosphonic acid (VPA) (97%) was purchased from TCI Ltd., UK. Acrylic acid (AA) (99%), 2,2′‐azobis(2‐methylpropionamidine)dihydrochloride (AAPH) (97%) and ethylene glycol diacrylate (EGDA) (90%) were all purchased from Sigma Aldrich Ltd., UK.

### Preparation of PVPA‐*co*‐AA hydrogels

The following method details the preparation of VPA‐30. Further details of the experimental conditions for all compositions are presented in Table 1. The concentration of initiator (AAPH) and crosslinker (EGDA) was kept constant in each case.

**Table 1:** Experimental Conditions for the Preparation of PVPA‐*co*‐AA Hydrogels (Where *n* is the Number of Moles and *V* is Volume)

Sample Code	Monomer Feed Ratio (VPA:AA)	*n*_VPA_ (mmol)	*V*_VPA_ (mL)	*n*_AA_ (mmol)	*V*_AA_ (mL)	*V*_H2O_ (mL)
VPA‐0	0:100	0.00	0.00	10.7	0.74	8.0
VPA‐10	10:90	1.07	0.08	9.67	0.67	6.0
VPA‐30	30:70	3.24	0.26	7.50	0.52	4.0
VPA‐50	50:50	5.37	0.42	5.37	0.37	2.0

VPA (0.35 g, 3.24 mmol), AA (0.54 g, 7.50 mmol), EGDA (3.5 mg, 0.21 mmol), and AAPH (2.9 mg, 0.01 mmol) were all dissolved in deionized water (4.0 cm^3^). The reactants were added together and the solution was purged with N_2_ for 20 min. The reaction mixture (400 μL) was aliquoted into a 24‐well plate with a well diameter of 15.6 mm. This was heated to 80°C for 30 min. The resulting gels were immersed in excess dH_2_O for 48 h to remove any unreacted monomer. The water was changed at intervals and the pH recorded. Purification of the hydrogels was considered as complete when the water attained a constant pH. Gels were produced in triplicate and were stored in dH_2_O before use.

### Characterization techniques

PVPA‐*co*‐AA hydrogels were dried fully (as verified by no change in weight) under vacuum and then ground into a fine powder. Elemental analyses were carried out using inductively coupled plasma mass spectrometry (ICP‐MS), by the School of Chemistry Microanalysis Service at the University of Manchester. The results are presented in Supporting Information Table S1.

Fourier transform infrared (FT‐IR) spectra were recorded using a Thermo Scientific Nicolet iS5 spectrometer with an iD5 diamond attenuated total reflectance (ATR) attachment over a wavenumber range of 4000–600 cm^−1^ and a resolution of 4 cm^−1^. The spectra were obtained from 16 scans.

### Swelling of hydrogels

Before swelling, gels were fully dried (as verified by no change in weight) in air in a 24‐well plate. The weight of the dry material (*W*_d_) was recorded. For the swelling experiment, 2 mL phosphate buffer (∼0.1 *M* Na_2_HPO_4_) was added to the well plate at pH 5.0, 7.3, or 9.0. The pH was adjusted using 0.1 *M* NaOH. The gels were left to swell for 24 h at 37°C. The supernatant was discarded and the gels were blotted with filter paper to remove any excess water. The weight of the swollen hydrogel (*W*_s_) was then recorded. Thus, the swelling was calculated using Eq. (1). The swelling experiment was repeated in triplicate for each hydrogel composition and the data were recorded as mean ± standard deviation.

(1) $$Swelling\;\left(\%\right)\;=\;\frac{W_{s}-\;W_{d}}{W_{d}}\;\times \;100$$

where *W*_s_ is the weight of the swollen hydrogel (g) and *W*_d_ is the weight of the dry material (g).

### Scanning electron microscope analysis

The as‐synthesized hydrogels were allowed to swell in deionized water for 24 h before being placed into a freezer, set at −80°C, for 1 h, to maintain the swollen structure of the gels. The frozen samples were subsequently freeze dried in an Edwards EF4 Modulyo vacuum freeze dryer (Thermo Fisher Scientific, UK). The freeze‐dried samples were mounted onto aluminum stubs using carbon tabs (Agar Scientific, UK) and then gold‐coated using an Emitech K550X sputter‐coater set at 40 mA for 1 min. The morphology of the hydrogels was then observed with a Zeiss EVO 50 field emission scanning electron microscope (SEM), using an acceleration voltage of 20.0 kV, a spot size of 400 and a working distance of 24.5 mm. Average pore size diameters were calculated using ImageJ software (https://imagej.nih.gov/ij/).

### Rheology

An ARES LN2 rheometer (TA Instruments, Hertfordshire, UK) with parallel‐plate geometry of 25 mm diameter was used for the rheological characterization of PVPA‐*co*‐AA hydrogels. Test methods of oscillatory strain sweep and frequency sweep were used. The tests were performed at a constant temperature (20.0°C) and a nominal gap of 2.5 mm. The strain sweep was performed at a frequency of 1.0 Hz while increasing the strain level from 1.0% to 100%. Samples were subjected to a steady strain ramp and the corresponding stress was measured (see Supporting Information Fig. S3). The linear viscoelastic region (LVR) from 5% to 15% was determined as a safe region without structural breakage from oscillatory strain.

The frequency sweep was performed at a constant strain of 10%, corresponding to a point in the middle of the LVR profile. The oscillatory frequency was increased from 0.1 to 10 Hz and the plots of storage (*G*′) and loss (*G*″) modulus against frequency were obtained using the manufacturer's supplied software. The complex viscosity (η*) was also plotted over the same frequency range.

### Cell culture

Human osteosarcoma derived osteoblast (SaOS‐2) cells were purchased from the European Collection of Authenticated Cell Cultures, UK (ECACC 89050205). Cells were cultured in McCoy's 5A medium (Sigma‐Aldrich) supplemented with 10% fetal bovine serum (FBS), antibiotics (100 U mL^−1^ penicillin, 100 mg mL^−1^ streptomycin) and 1% l‐glutamine. Culture medium was replenished every 24 h. Hydrogels were placed on glass coverslips (13 mm diameter, Scientific Laboratory Supplies) in 24‐well plates and sterilized using UV‐irradiation for 1 h. Cells were counted and seeded onto scaffolds at a density of 50,000 cells/cm^2^. The volume of seeding media was minimized to ensure that the maximum number of cells would be in contact with the top surface of the hydrogel. Once adhered, the well was flooded with excess media. Plates were cultured at 37°C and 5% CO_2_ for up to 14 days.

### Live/Dead® staining

Live/Dead® staining solution (Invitrogen, UK) was prepared by adding ethidium homodimer‐1 (4 μ*M*) and calcein‐AM (2 μ*M*) to sterile phosphate buffered saline (PBS). Cells were cultured on the various hydrogels and glass coverslips were used as a control. At certain time points (4, 24, and 72 h), the culture medium was removed from the samples and the wells were washed with sterile PBS. The prepared staining solution (150 μL) was added to each well and incubated for 30 min at 37°C and 5% CO_2_. The hydrogels were then mounted onto glass microscope slides and viewed under the Nikon Eclipse 50i fluorescence microscope with a camera attachment. Cell spreading and proliferation was quantified using ImageJ software and the results are presented in Supporting Information Figure S4.

### Determination of cell number

Cells were cultured on the various hydrogels and tissue culture plastic (TCP) was used as a control. At certain time points (1, 3, 7, and 14 days), the culture medium was removed and samples were washed with sterile PBS. The samples were immersed in 1 mL lysis buffer (0.1% Triton X‐100 in dH_2_O) and three freeze–thaw cycles were performed. The PicoGreen® dsDNA stain solution was prepared as outlined by the manufacturer (ThermoFisher Scientific, UK). One hundred microliters of each sample and 100 μL of the PicoGreen® stain solution were added to a flat‐bottomed 96‐well plate (Nunc, UK). The fluorescence was measured at an excitation of 485 nm and an emission at 520 nm using a Fluostar Optima Fluorescence Microplate Reader (BMG Labtech, Germany). A calibration curve was produced prior to the measurement using a set of dsDNA standards (0–2 μg mL^−1^), prepared in TE (Tris EDTA) buffer. The cell number was quantified by means of the calibration curve (*R* ^2^ > 0.999) (see Supporting Information Fig. S5).

### Cell metabolic activity

Metabolic activity was determined using the AlamarBlue® assay. Cells were cultured on the various hydrogels and TCP was used as a control. At certain time points (1, 3, 7, and 14 days) the culture medium was removed and samples were washed with sterile PBS. One milliliter of fresh culture medium was added along with 100 µL of AlamarBlue® solution (8.0 mg mL^−1^ resazurin salt in PBS). The plates were incubated at 37°C and 5% CO_2_ for 4 h. The fluorescence was then measured at an excitation of 544 nm and an emission at 590 nm using a Fluostar Optima Fluorescence Microplate Reader (BMG Labtech). The fluorescence was recorded as a function of dsDNA content (measured using the PicoGreen® assay) to give an indication as to the metabolic activity relative to the number of cells (see Supporting Information Fig. S6).

### Statistical analysis

Statistical evaluation of data was performed using the GraphPad Prism™ software package. Tests were carried out in triplicate (*n* = 6) and all data are reported as mean ± standard deviation at a significance level of *p ≤ 0.05, **p ≤ 0.01, ***p ≤ 0.001. The data were tested for normality and a one‐way analysis of variance (ANOVA) was then carried out with the Tukey test to compare the groups of the SaOS‐2 cell culture.

## RESULTS

### Synthesis and characterization of PVPA‐*co*‐AA hydrogels

**Figure 1:**
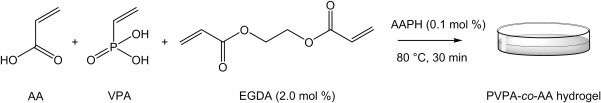
Production of PVPA‐*co*‐AA hydrogel from AA and VPA monomers, using EGDA as a crosslinking agent.

The copolymerization of AA and VPA has been investigated in detail in previous work.^26^ Similar reaction conditions were used for the production of PVPA‐*co*‐AA hydrogels (Fig. 1). EGDA was utilized as a difunctional crosslinking agent, since it has previously been shown to be successful in the production of PVPA hydrogels.^28^ The polymerization was carried out at 80°C for 30 min, with a crosslinker concentration of 2.0 mol %, to achieve the optimum structural and mechanical properties of the hydrogels (see Supporting Information Figs. S1 and S2).

Hydrogels were produced with VPA monomer feed contents of 0, 10, 30, and 50 mol % and these were characterized by elemental analysis (see Supporting Information Table S1). The copolymer ratio (VPA:AA) in the hydrogels differed slightly from the monomer feed ratios due to the lower reactivity of VPA when compared with AA.^26^

**Figure 2:**
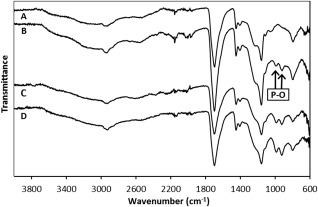
FT‐IR spectra of (A) VPA‐0, (B) VPA‐10, (C) VPA‐30, and (D) VPA‐50.

The hydrogels were also characterized by FT‐IR spectroscopy (Fig. 2). It can be observed that all hydrogels exhibit characteristic signals between 2800 and 2400 cm^−1^, representing the O—H stretch of the AA and phosphonic acid side groups. The C—H stretch and bend signals are observed at 3000–2800 and 1500–1375 cm^−1^, respectively.

The strong band at 1696 cm^−1^ was attributed to the C=O stretch of the AA side group. Furthermore, the signals which are observed at 1300–1050 cm^−1^, of medium‐strong intensity, denote the C—O stretch of the same group. When VPA is incorporated into the hydrogels, as in the spectra of (B), (C), and (D), two new bands appear between 1090 and 905 cm^−1^, which represent the P—O stretch of the phosphonic acid side group. These signals increase in intensity as the VPA content in the feed is increased. Therefore, this provides strong evidence for the successful synthesis of PVPA‐*co*‐AA hydrogels, with increasing VPA contents.

### Swelling of hydrogels

The swelling behavior of hydrogels in response to external stimuli, such as pH and ionic strength, can give an indication as to their effectiveness in biomaterials applications. Therefore, in this study, the swelling of the hydrogels was determined gravimetrically in phosphate buffer solutions of pH 5.0, 7.3, and 9.0.

**Figure 3:**
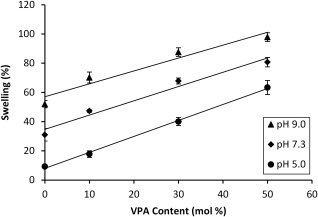
Swelling of PVPA‐*co*‐AA hydrogels as a function of VPA content, at pH 5.0, 7.3, and 9.0. Lines are added as a guide to the eye.

As can be seen in Figure 3, the swelling increased with an increase in VPA content in the hydrogels. This can be attributed to the properties of the respective homopolymers. Both PAA and PVPA are negatively charged polyelectrolytes; however, whereas PAA is a weak polyelectrolyte (p*K*_a_ ∼ 4.50),^29^ PVPA has been described as medium‐strong (p*K*_a_ ∼ 2.75).^30^ This means that there is greater dissociation of phosphonic acid groups in aqueous media, resulting in enhanced electrostatic repulsions within the polymer network and hence an increase in the swelling of the gels.

An increase in the pH of the buffer solution also resulted in greater swelling capacities (Fig. 3) due to the enhanced ionization of carboxylic and phosphonic acid groups. Thus, increased electrostatic repulsions occur, which leads to greater swelling of the hydrogels. The pH was adjusted using 0.1 *M* NaOH. Therefore, as the pH is increased, the ionic strength of the solution will also increase. Greater charge shielding will occur, which could reduce the effective swelling of the hydrogels. However, this did not affect the overall trend in the swelling capacities of hydrogels with increasing pH of the solution and so was not thought to be significant.

### SEM analysis

**Figure 4:**
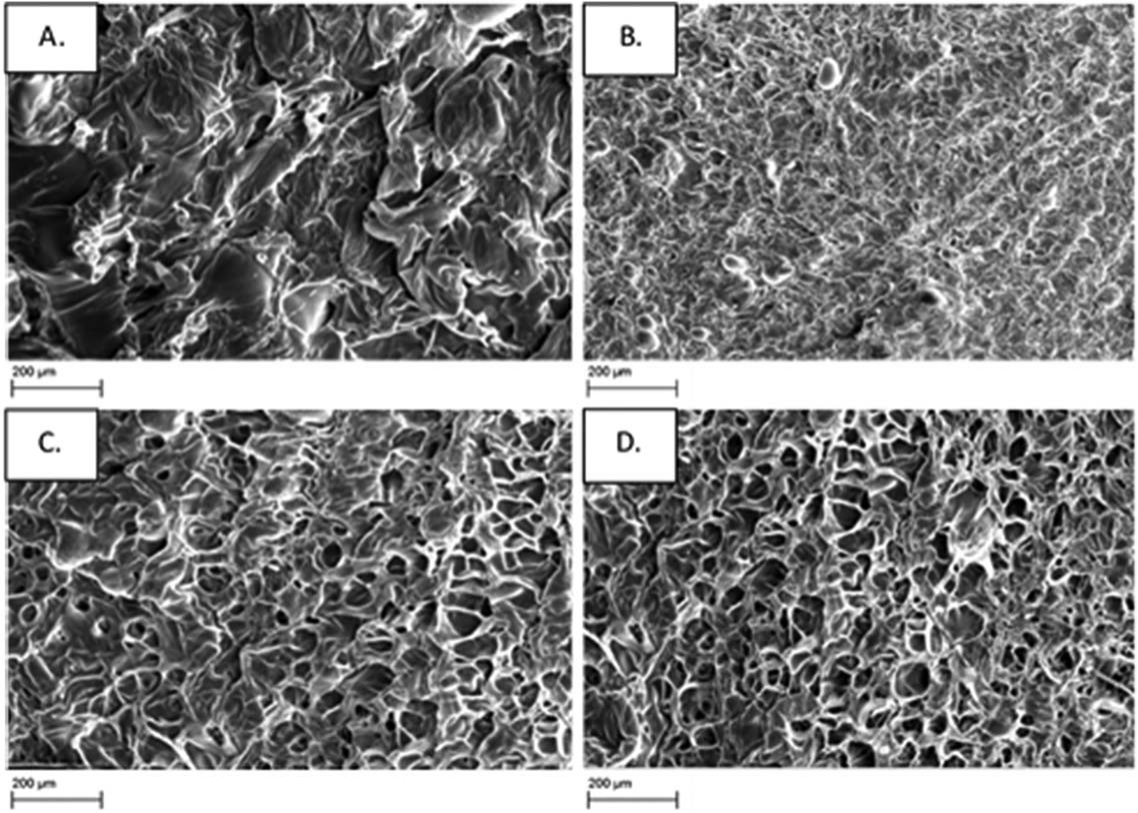
SEM images to show the difference in morphology of freeze‐dried PVPA‐*co*‐AA hydrogels with increasing VPA contents. (A) VPA‐0, (B) VPA‐10, (C) VPA‐30, and (D) VPA‐50.

PVPA‐*co*‐AA hydrogels were freeze‐dried and then investigated using a SEM. A difference in their physical morphology can be observed with different monomer feed ratios (Fig. 4). It can be seen that the freeze‐dried hydrogel composed of PAA (VPA‐0) has a flaky structure, whereas the addition of low amounts of VPA, such as in VPA‐10, produced a porous structure with an average pore diameter of 49 ± 20 μm. Freeze‐dried hydrogels with higher VPA contents formed structures with larger pores and an interconnected pore network. For example, VPA‐30 hydrogels had an average pore diameter of 84 ± 37 μm and for VPA‐50, the average diameter was 100 ± 32 μm.

### Rheological properties of PVPA‐*co*‐AA hydrogels

**Figure 5:**
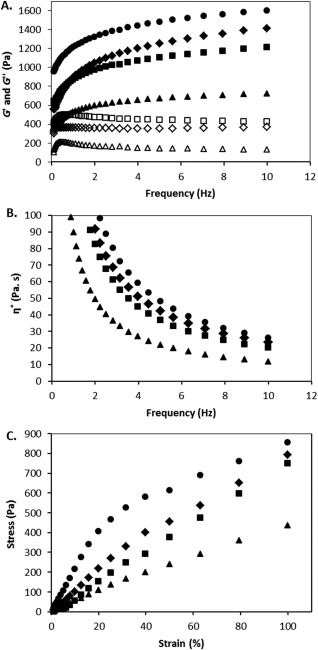
(A) Storage (*G*′) and loss (*G*″) modulus of PVPA‐*co*‐AA hydrogels across a frequency sweep of 0.1–10 Hz (*G*′ is represented by closed symbols and *G*″ by open symbols), (B) complex viscosity (η*) of PVPA‐*co*‐AA hydrogels across a frequency sweep of 0.1–10 Hz, and (C) stress–strain curve for PVPA‐*co*‐AA hydrogels, with increasing VPA contents, across a strain range of 1–100%, for VPA‐0 (circles), VPA‐10 (diamonds), VPA‐30 (squares) and VPA‐50 (triangles). All measurements were performed at 20.0°C.

Another important property of hydrogels is their rheological behavior. Figure 5A shows the change in storage (*G*′) and loss (*G*″) modulus for PVPA‐*co*‐AA hydrogels with increasing frequency of oscillation. Figure 5B shows the change in complex viscosity across the same frequency range. It was found that an increase in VPA content in the hydrogels led to a large decrease in *G*′ and a general increase in *G*″, indicating the dominance of viscous fluid behaviors in gels with high VPA contents. This behavior is expected since the water content increases proportional to the amount of VPA in the hydrogels.

The complex viscosities of the hydrogels all show a decrease with an increase in frequency, which indicates pseudoplastic behavior of the materials. This decrease in η* represents a deformation of the hydrogel structure at high frequencies of shear strain.

Figure 5C shows the stress‐strain curves for hydrogels with increasing VPA contents. From this graph, the yield stress can be determined as the point at which the slope of the curve changes and the material is permanently deformed. The yield stress for VPA‐0 can be seen at a strain of 20%, whereas for VPA‐10 this occurs at approximately 40% strain. For VPA‐30 and VPA‐50, the yield stress point is not reached within the strain range used for this experiment. This implies that hydrogels with higher VPA contents are more ductile and thus able to resist a very high strain before failure. The change in *G*′ and *G*″ with increasing strain is presented in Supporting Information Figure S3 for all of the PVPA‐*co*‐AA hydrogels. The crossover point gives an indication as to the yield strain of the material and this point increases with increasing VPA content, which corroborates the data presented in Figure 5C.

### Cell proliferation and metabolic activity

**Figure 6:**
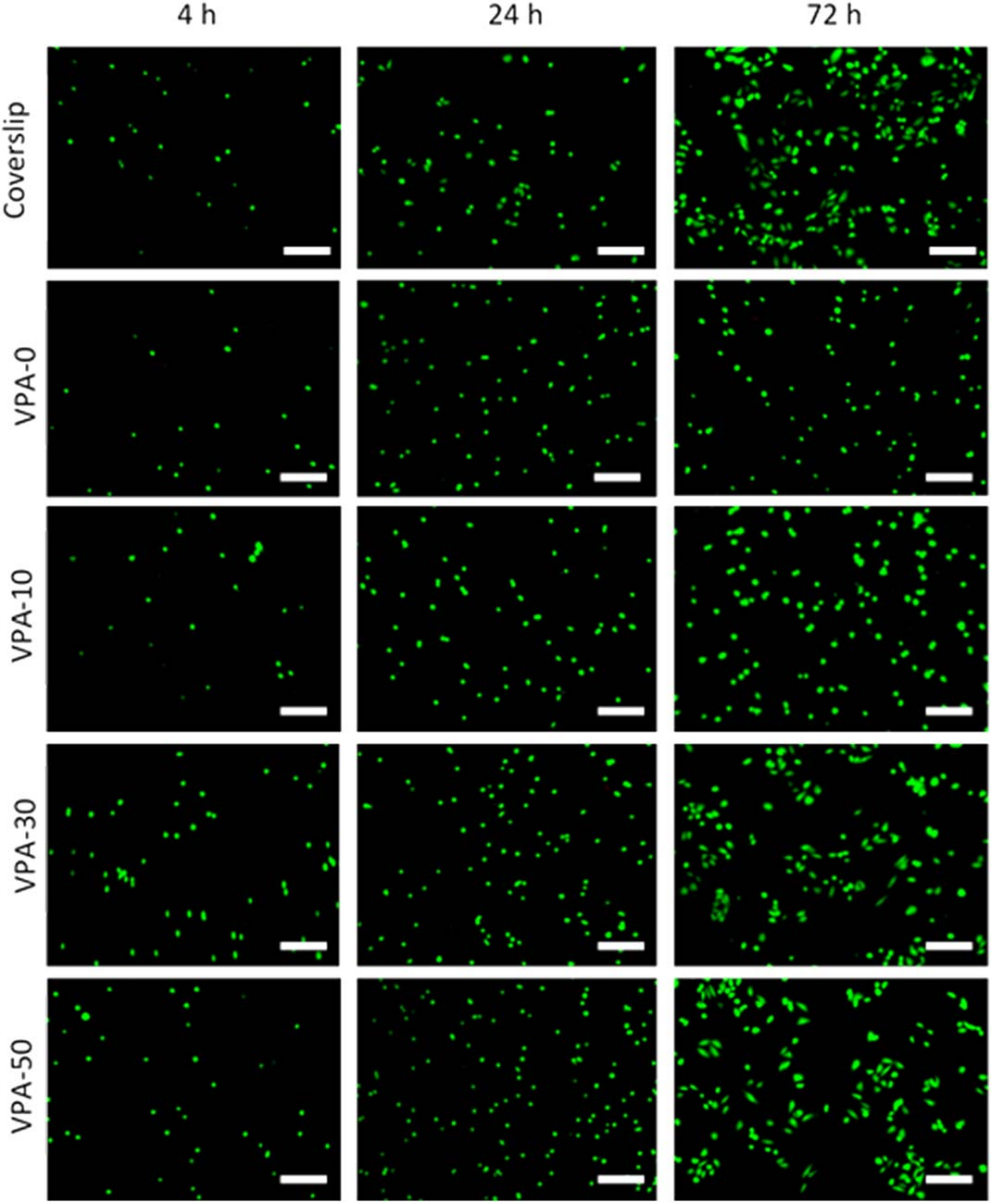
Live/Dead® imaging of human osteoblast cells to illustrate cell viability on PVPA‐*co*‐AA hydrogels, as a function of copolymer composition, over 72 h. Glass coverslips were used as a control. Live cells stained green, dead cells stained red. *n* = 6, scale bar 100 μm.

The effect of VPA content on the osteoblast response to PVPA‐*co*‐AA hydrogels was investigated. Live/Dead® cell staining was used to study osteoblast viability for up to 72 h of cell culture (Fig. 6). The attachment and proliferation of live (green) SaOS‐2 cells can be visualized on all hydrogels, with a lack of dead (red) cells. This confirms the cytocompatibility of the PVPA‐*co*‐AA hydrogels with the SaOS‐2 cell line, which was not affected by copolymer composition.

The density and morphology of the growing cells differed depending on the VPA content in the gels. Quantification of cell density and the morphology of each cell (cell spreading) is presented in Supporting Information Figure S4. A general increase in cell proliferation was found on hydrogels with higher VPA contents. Furthermore, VPA‐0 and VPA‐10 hydrogels showed little cell spreading, whereas cells seeded onto VPA‐30 and VPA‐50 gels demonstrated enhanced spreading morphologies after 72 h in culture medium. At this point, cell spreading was comparable to that of the control. The increase in cell adhesion, proliferation and spreading on hydrogels with higher VPA contents can be attributed to the increase in their swelling and porosity. If a material has a nonfibrous, porous architecture with larger pores, cells may stretch along the walls of a pore. However, cells may not adopt such a spreading, spindle‐shaped morphology on materials that are closely packed with smaller pores. Furthermore, the increased swelling of hydrogels allows for the infiltration of cells through the structure and more efficient transport of nutrients to the cells.

**Figure 7:**
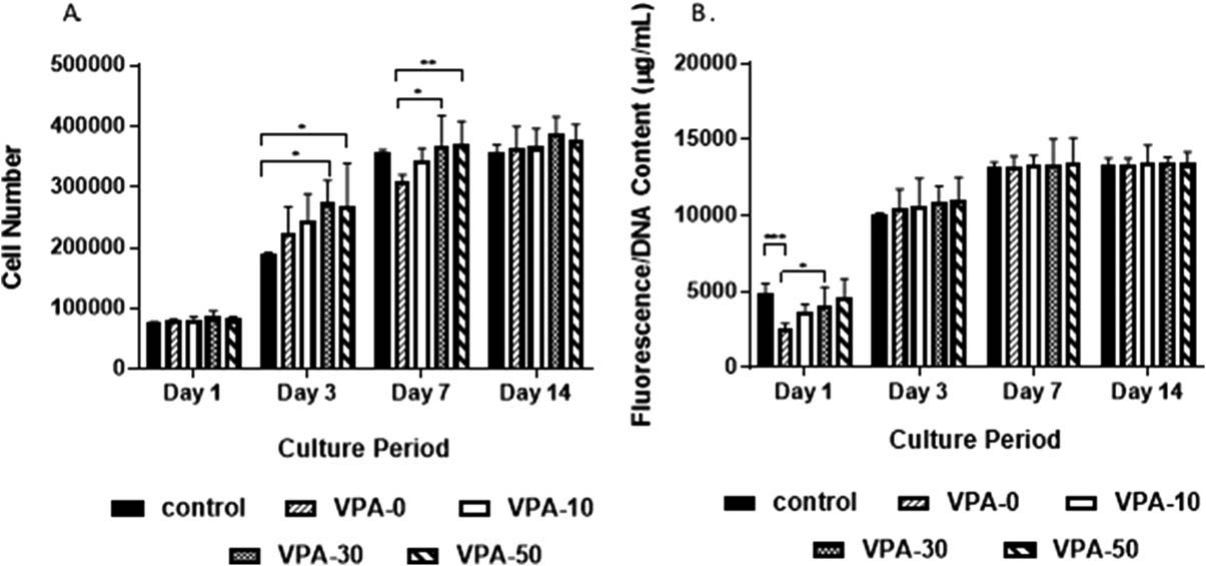
(A) Osteoblast proliferation and (B) osteoblast metabolic activity on PVPA‐*co*‐AA hydrogels, with increasing VPA content, over 14 days. Mean ± SD, *n* = 6 triplicates (*p ≤ 0.05, **p ≤ 0.01, ***p ≤ 0.001).

The increase in cell adhesion was confirmed in Figure 7A, which shows an increase in cell number as a function of VPA content, up to 7 days of culture. By day 14, the highest cell number was observed on VPA‐30 gels, although the difference between hydrogels was not statistically significant (*p* > 0.05).

The metabolic activity of SaOS‐2 cells, seeded onto PVPA‐*co*‐AA hydrogels, was measured using the AlamarBlue® assay. The data are presented in Figure 7B as a function of dsDNA concentration, calculated using the PicoGreen® assay. This gives an indication of the metabolic activity relative to the number of cells present. The raw fluorescence data are shown in Supporting Information Figure S6. At day 1, there was a clear increase in cell metabolic activity with higher VPA contents. However, at days 3, 7, and 14, there was no significant difference between the different hydrogels. This indicates that the cell metabolic activity is proportional to the cell number. After 3 days of culture, the hydrogels have no significant effect on cell metabolic activity.

## DISCUSSION

PVPA‐*co*‐AA has been identified as a potential candidate for use as a bone graft substitute, owing to its ability to bind to calcium ions *in vivo* and thus promote mineralization and bone formation.

We have reported the synthesis and characterization of PVPA‐*co*‐AA hydrogels, with a range of VPA feed contents. Previously, we have investigated the solution polymerization of PVPA‐*co*‐AA and have demonstrated the much lower reactivity of VPA when compared with AA,^26^ which leads to low incorporation of VPA into the final copolymer. This was also found to be the case for the preparation of hydrogels of PVPA‐*co*‐AA, as confirmed by elemental analysis. A discrepancy was found between the VPA content in the monomer feed and in the hydrogel product. However, this difference was relatively small and the VPA content in the hydrogels was shown to increase as the VPA content in the monomer feed was increased. This was confirmed using FT‐IR spectroscopy, whereby the P—O stretch of the phosphonic acid group (1090–905 cm^−1^) increased in intensity with greater VPA feed contents. These peaks were absent from the spectrum of VPA‐0, which only contained AA.

The swelling properties of polyelectrolyte hydrogels is one of the main driving forces for cell migration. The swelling of PVPA‐*co*‐AA hydrogels was found to increase with an increase in VPA content. This was attributed to the greater acidity, and hence greater degree of dissociation in aqueous media, of VPA when compared with AA. In addition, VPA has been shown to be more hydrophilic than AA, as demonstrated by a reduction in water contact angle (see Supporting Information Figure S7 and Table S2). Therefore, hydrogels with higher VPA contents are more likely to have a high water uptake.

It is hypothesized that a greater degree of swelling will result in optimal cell infiltration and transport of nutrients, waste products and growth factors.^31^, ^32^ Furthermore, the degree of ionization of the hydrogel may affect the transport and adsorption of charged molecules, such as proteins. López‐Pérez et al.^33^ have shown that VPA can attract positively charged proteins from cell culture media. This was found to enhance SaOS‐2 cell adhesion and proliferation. Therefore, it is expected that the hydrophilic surface of PVPA‐*co*‐AA hydrogels, coupled with their strong negative charge, can allow the adsorption of positively charged ECM proteins. This, in turn, may lead to greater osteoblast adhesion and proliferation.

**Figure 8:**
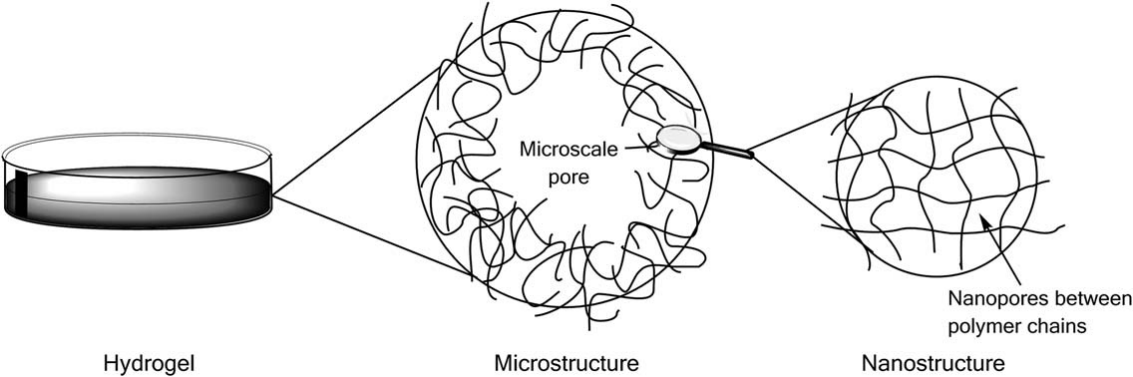
Schematic representation of the hierarchical structure of hydrogels with both micro‐ and nanoscale pores.

The increased swelling of hydrogels with higher VPA contents, as a result of enhanced electrostatic repulsions, should lead to a greater pore size within the polymer network. The morphology of the freeze‐dried PVPA‐*co*‐AA hydrogels was observed under SEM (Fig. 4). It was shown that VPA‐0 had a flaky structure and VPA‐10 contains few small pores. However, VPA‐30 and VPA‐50 contain much larger, microscale pores. It is proposed that a hierarchical structure exists within the hydrogels, which is depicted in Figure 8. The hydrogel structure consists of crosslinked polymer chains with nanoscale pores between the chains, which allow the infiltration of water. As the hydrogels swell, there is an increase in the size of the nanopores. This effect is greater for gels that contain higher VPA contents, owing to the increased acidity and hydrophilicity of VPA when compared with AA.

However, microscale pores are also observed in the structure of freeze‐dried hydrogels that contain 30 and 50 mol % VPA. It is suggested that the large difference in reactivity between AA and VPA^26^ may account for these defects. AA is much more reactive than VPA and so, during the early stages of polymerization, AA and EGDA can react rapidly to produce AA rich regions with a high crosslink density. As the AA becomes fully consumed, VPA homopolymerization can occur. Pendant EGDA groups may react with VPA during this stage, resulting in lightly crosslinked regions with inhomogeneously distributed crosslinks and dangling chain ends. This would potentially lead to regions that can swell almost indefinitely but are constrained by the surrounding network. This may account for the microscale pores or voids found in the freeze‐dried samples of VPA‐30 and VPA‐50.

The porosity and pore architecture of hydrogels used in bone tissue engineering play a significant role in cell adhesion, migration, and proliferation. A high degree of porosity is required for angiogenesis to occur, which is a key requirement for vascularized tissue. Furthermore, the extent of ECM secretion increases by increasing the pore size within the microstructure of three‐dimensional scaffolds.^34^ It has been noted that a pore size of 100–350 μm is ideal for bone regeneration.^35^ Therefore, it can be hypothesized that VPA‐30 and VPA‐50 will be the most effective for this application owing to their structure and large average pore diameter. Although there are differences between the freeze‐dried sample and swollen hydrogels, the pore size is likely to be greater in VPA‐30 and VPA‐50 gels when swollen in cell culture media. Furthermore, the porous structure of these gels can be adjusted to mimic the architecture of bone (particularly the porous nature of cancellous bone).

Natural bone is both porous and mechanically durable. Therefore, when designing bone graft substitutes, it is important to have high porosities and swelling characteristics as well as sufficient mechanical integrity to provide a support structure. It was found that hydrogels with lower VPA contents, that is, VPA‐0 and VPA‐10, had a greater mechanical strength, which was attributed to their higher degree of crosslinking. VPA‐30 and VPA‐50 gels demonstrated more viscous fluid behavior as a result of their greater swelling capacities and highly porous structure (Fig. 8). This implies that VPA‐0 hydrogels would be more suitable for load‐bearing applications. However, VPA‐30 and VPA‐50 gels were shown to be more ductile and could resist large amounts of strain without permanent deformation of their structure. This is desirable for clinical applications where they can be molded into a bone defect site to be used as a bone void filler. A stronger, stiffer material can then be used as a support structure to withstand the mechanical forces exerted by the body.

An increase in SaOS‐2 cell adhesion and proliferation was observed on hydrogels with 30 and 50 mol % VPA. It was found that rapid proliferation of the cells occurred on these hydrogels, with cells becoming confluent on all hydrogels after 14 days of culture. Additionally, cells that were seeded onto VPA‐30 and VPA‐50 hydrogels demonstrated superior spreading morphologies, comparable to that of the control. This was attributed to the increase in swelling and porosity of these gels, which, as described previously, can lead to enhanced protein adsorption to facilitate cell attachment and proliferation.

The metabolic activity of the SaOS‐2 cells was measured relative to the number of cells. It was shown that there was no significant difference between hydrogels with different VPA contents, which indicates that the cell metabolic activity is proportional to the cell number. Therefore, the results demonstrate that hydrogels with higher VPA contents lead to enhanced cell adhesion and proliferation without having any significant effect on cell metabolic activity.

## CONCLUSIONS

In this study, hydrogels of PVPA‐*co*‐AA have been produced and evaluated for their ability to promote osteoblast adhesion and proliferation. An increase in SaOS‐2 cell adhesion and proliferation was observed as a function of VPA content in the hydrogels. In addition, cells that were seeded onto VPA‐30 and VPA‐50 hydrogels demonstrated superior cell spreading morphologies. This was attributed to the greater swelling and increase in porosity of hydrogels with higher VPA contents. It was also found that cell metabolic activity increased proportionally to cell number and so it can be concluded that the hydrogels had no significant effect on cell metabolic activity.

VPA‐30 and VPA‐50 hydrogels were shown to be more flexible and could be deformed to large extents without permanent deformation of their structure. Therefore, it is proposed that these gels are most suitable for use in clinical settings where they can be molded into a bone defect site.

This work suggests that hydrogels with 30 or 50 mol % VPA are ideal for use as bone void fillers. The structures of these gels encourage high swelling and increased osteoblast‐like cell attachment and proliferation. Furthermore, this work shows that the mechanical and cell adhesion properties of the gels can be tuned by altering the copolymer composition.

## Supporting information

Supporting Information
