# Supplementary material for: Poly(vinylphosphonic acid‐co‐acrylic acid) hydrogels: The effect of copolymer composition on osteoblast adhesion and proliferation
Source: J Biomed Mater Res A. 2017 Oct 24;106(1):255–64. doi: 10.1002/jbm.a.36234 (PMC5725815; doi:10.1002/jbm.a.36234)
Supplement: Supplementary file 1 — Supporting Information [file JBM-106-255-s001.docx]

Supporting Information

Poly(vinylphosphonic acid-*co*-acrylic acid) hydrogels: The effect of copolymer composition on osteoblast adhesion and proliferation

**Rebecca E. Dey,^1^ Ian Wimpenny,^2^ Julie E. Gough,^2^ David C. Watts,^3^ and Peter M. Budd^1^***

^1^School of Chemistry, The University of Manchester, Manchester, M13 9PL, U.K.

^2^School of Materials, The University of Manchester, Manchester, M13 9PL, U.K.

^3^School of Medical Sciences and Photon Science Institute, The University of Manchester, Manchester, M13 9PL, U.K.

page

1. Elemental analysis of PVPA-*co*-AA hydrogels 2
2. Rheological Properties of PVPA-*co*-AA hydrogels 3

Effect of crosslinker concentration 3

Effect of polymerisation time 4

Effect of monomer feed content 5

1. Osteoblast proliferation and metabolic activity 6

Quantification of cell spreading and proliferation 6

Calibration curve for the calculation of cell number 7

Calculation of cell metabolic activity 8

1. Water contact angle measurements 9
2. **Elemental analysis of PVPA-*co*-AA hydrogels**

Elemental analysis data from ICP-MS is shown in Table S1.

Table S1. Elemental analysis data of PVPA-*co*-AA hydrogels with different feed compositions.

| **Sample Code** | **Monomer Feed Ratio (VPA:AA)** | **C (%)** | **H (%)** | **P (%)** | **Copolymer Ratio (VPA:AA)** |
| --- | --- | --- | --- | --- | --- |
| VPA-0 | 0:100 | 46.8 | 6.09 | 0.00 | 0:100 |
| VPA-10 | 10:90 | 44.1 | 5.96 | 2.60 | 7:93 |
| VPA-30 | 30:70 | 36.4 | 5.91 | 8.08 | 24:76 |
| VPA-50 | 50:50 | 31.2 | 5.89 | 12.6 | 41:59 |

The mole ratio, *r*_P/C_, of P to C in the copolymer was calculated from the elemental analysis data using equation S1:

$$r_{P/C}= \frac{\%P \times M_{C}}{\%C \times M_{P}} (S1)$$

where *M*_C_ is the molar mass of carbon and *M*_P_ is the molar mass of phosphorus. The mole fraction, *x*_VPA_, of VPA in the copolymer was then calculated using equation S2:

$$x_{\mathrm{VPA}}= \frac{1}{1+(\frac{1-2r_{P/C}}{3r_{P/C}})} (S2)$$

1. **Rheological properties of PVPA-*co*-AA hydrogels**

Figure S1A shows the effect of EGDA concentration on the swelling of the hydrogels. The VPA content was maintained at 30 mol % in each case and the polymerisation was carried out for 30 min. It was found that the swelling decreased with an increase in crosslinker concentration. This was accompanied by an increase in the storage modulus (*G*’) as shown in Figure S1b, which represents a reinforced crosslinked network. For applications in bone tissue scaffolds, it is important for the material to have a relatively high mechanical strength. However, a high porosity and a large degree of swelling are vital for cell infiltration and the transport of nutrients. Therefore, an EGDA concentration of 2.0 mol % was chosen for the production of hydrogels with a good mechanical strength and a reasonable degree of swelling.


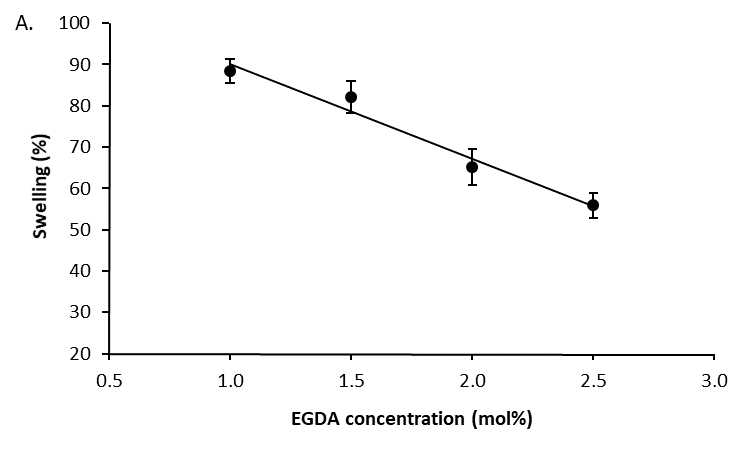

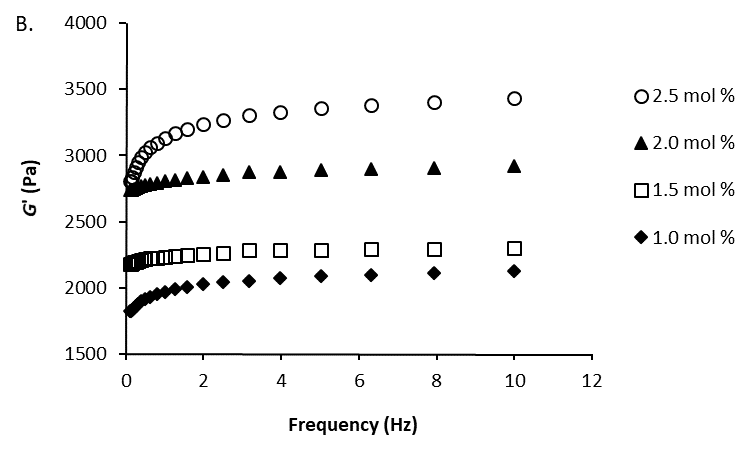


**Figure S1.** (A) Effect of crosslinker (EGDA) concentration on the dynamic swelling of PVPA-*co*-AA hydrogels in phosphate buffer solution at pH 7.3. (B) Effect of EGDA concentration on the storage (*G*’) modulus of PVPA-*co*-AA hydrogels across a frequency range of 0.1 to 10 Hz. In each case, the VPA content was 30 mol %.

Figure S2A shows the effect of polymerisation time on the dynamic swelling of PVPA-*co*-AA hydrogels. The VPA content was 30 mol % in each case. It can be observed that the swelling of the gels decreases with longer polymerisation times. This can be attributed to the increase in the degree of polymerisation with time, which leads to a more highly crosslinked structure. Therefore, it follows that there is a general increase in the storage modulus (*G*’) with polymerisation time (Figure S2B). Again, a balance must be found between a high mechanical strength of the gels and a high degree of swelling. Therefore, a polymerisation time of 30 min was chosen to obtain hydrogels with optimal properties for cell adhesion.


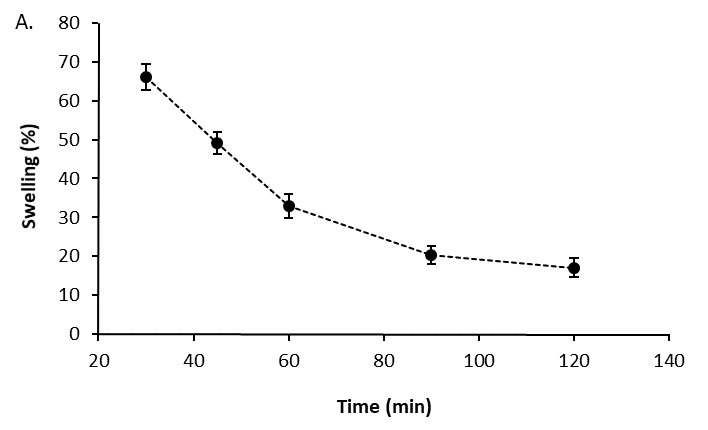


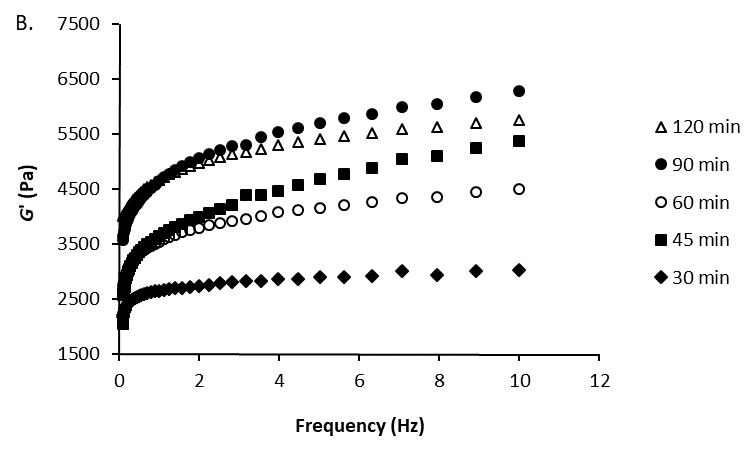


**Figure S2.** (A) Effect of polymerisation time on the dynamic swelling of PVPA-*co*-AA hydrogels in phosphate buffer solution at pH 7.3. (B) Effect of polymerisation time on the storage modulus (*G*’) of PVPA-*co*-AA hydrogels across a frequency range of 0.1 to 10 Hz. In each case, the VPA content was 30 mol %.

Figure S3 shows the change in storage (*G*’) and loss (*G*’’) modulus with increasing strain for VPA-0 (A), VPA-10 (B), VPA-30 (C) and VPA-50 (D). The *G*’ and *G*’’ crossover point, where viscous fluid behaviours start to dominate over elastic behaviour occurs at a strain rate of 60% for VPA-0 and 80% for VPA-10. For VPA-30 and VPA-50 hydrogels, this point is not reached within the strain range of this experiment. This indicates that hydrogels with lower or no VPA content are more brittle, with their crosslinked structure being broken down under lower values of strain. VPA-30 and VPA-50 hydrogels are more flexible and are thus able to resist high values of strain before failure.


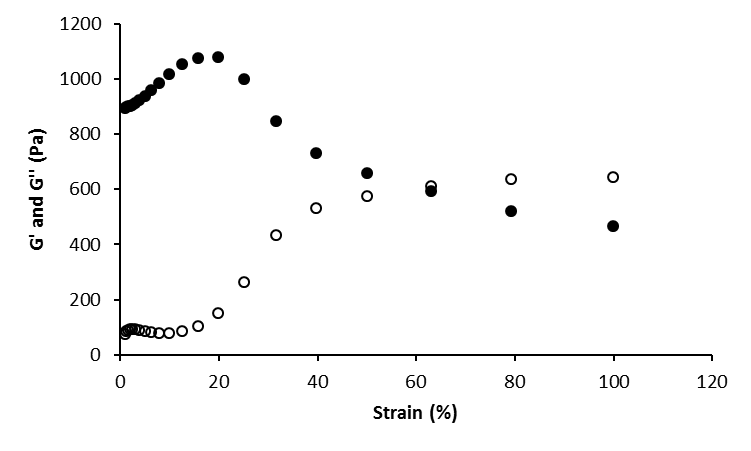

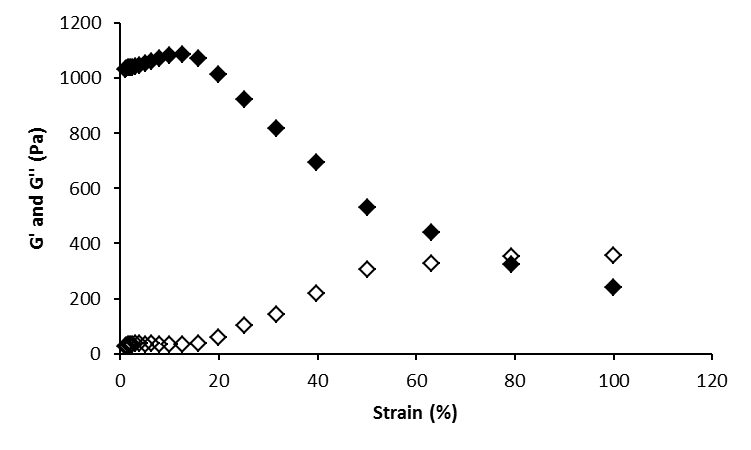

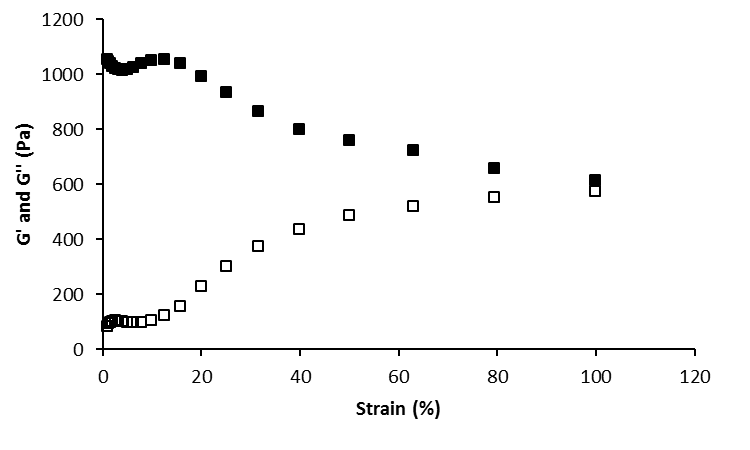

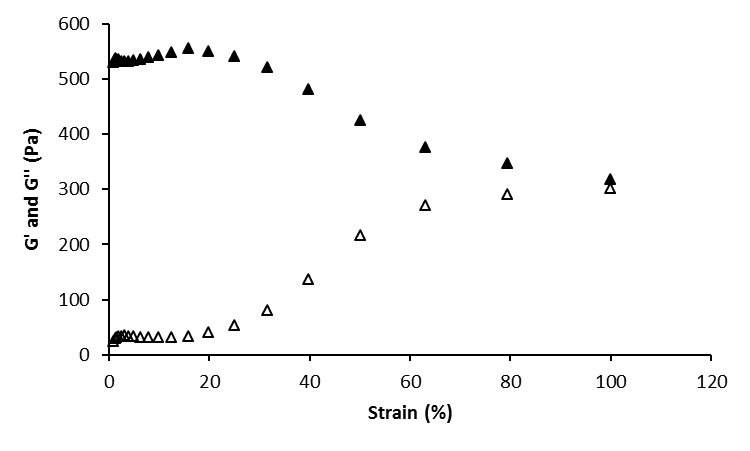


A.

B.

C.

D.

Figure S3. Storage (*G*’) and loss (*G*’’) modulus of PVPA-*co*-AA hydrogels, with increasing VPA contents, across a strain range of 1-100%. (A) VPA-0, (B) VPA-10, (C) VPA-30 and (D) VPA-50. *G*’ is represented by closed symbols and *G*’’ is represented by open symbols.

1. **Osteoblast proliferation and metabolic activity**

**Quantification of cell spreading and proliferation**

Figure S4 shows the cell density (cell/mm^2^) and average cell area (μm^2^) of SaOS-2 cells seeded onto PVPA-*co*-AA hydrogels, quantified using ImageJ analysis. Figure S4A shows that there is a general increase in cell proliferation with increasing VPA content. This effect is more pronounced at 4 h and 24 h. At 4 h, VPA-30 shows the highest proliferation which demonstrates that the cells initially grow more rapidly on this substrate. These results corroborate the increase in cell number observed using the PicoGreen® assay.

Furthermore, enhanced cell spreading is observed on hydrogels with higher VPA contents (Figure S4B). At 4 h and 24 h, there is no significant difference in cell spreading between hydrogels with different compositions. However, at 72 h, a clear increase in cell area can be seen, with an increase in VPA content resulting in increased cell spreading.


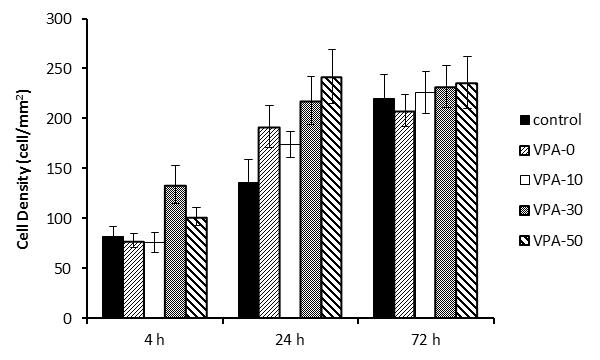

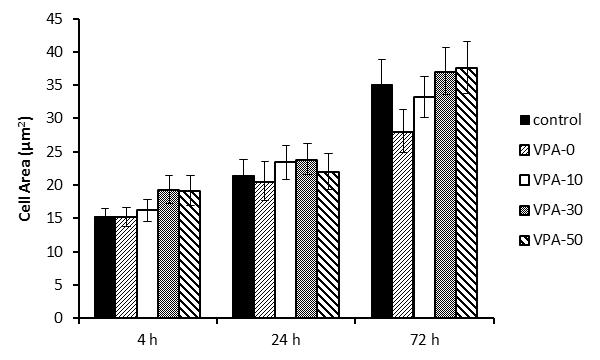


A.

B.

Figure S4. (A) Osteoblast proliferation and (B) cell spreading on PVPA-*co*-AA hydrogels, with increasing VPA content, over 72 h. Mean ± SD, *n* = 9 triplicates.

**Calibration curve for the calculation of cell number**

Figure S5 shows the calibration curve for the determination of dsDNA concentration of cells seeded onto PVPA-*co*-AA hydrogels. The PicoGreen® dsDNA stain solution (100 μL) was added into solutions of DNA standards (100 μL) at different concentrations (0-2 μg mL^-1^), prepared in TE buffer, and the corresponding fluorescence intensity was recorded.


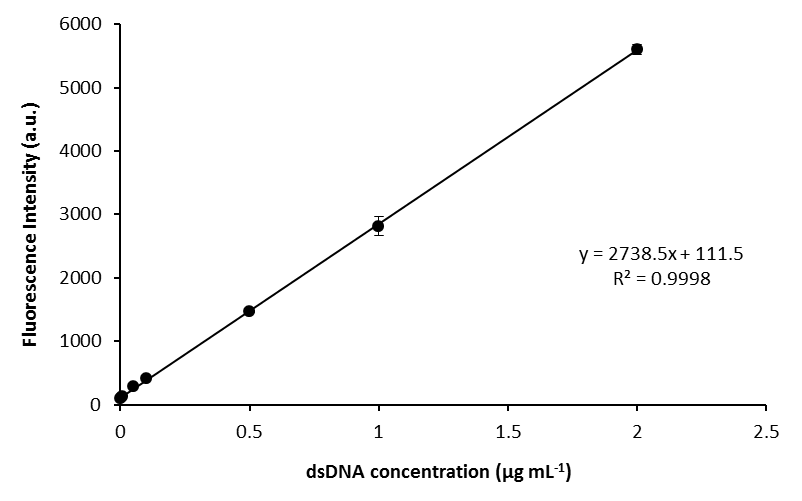


Figure S5. Calibration curve for the determination of dsDNA concentration of cells seeded onto PVPA-*co*-AA hydrogels.

The PicoGreen® stain solution (100 μL) was then added to 100 μL of the sample solution and the fluorescence intensity was recorded. The dsDNA concentration was then calculated using the calibration curve and equation S3:

$$dsDNA concentration \left( \mu g mL^{-1} \right)= \frac{Fluorescence Intenisty-111.5}{2738.5} (S3)$$

The cell number could then be estimated by assuming that each cell contains 5 pg dsDNA[^1^](#_ENREF_1) and using equation S4:

$$Cell number= \frac{M_{\mathrm{dsDNA}}}{5 \times{10}^{-6}} (S4)$$

where *M*_dsDNA_ is the mass of dsDNA (μg) in 1 mL lysis buffer.

**Calculation of cell metabolic activity**

Cell metabolic activity was measured using the AlamarBlue® assay. Figure S6 shows the fluorescence intensity of cells seeded onto PVPA-*co*-AA hydrogels, with increasing VPA content, over 14 days. This gives an estimation of the average metabolic activity of the cells on the hydrogels.

Figure S6. Fluorescence Intensity (measured using the AlamarBlue® assay) of cells seeded onto PVPA-*co*-AA hydrogels, with increasing VPA contents, over 14 days. Mean ± SD, *n* = 9. (**p* ≤ 0.05, ***p* ≤ 0.01 and ****p* ≤ 0.001).

The metabolic activity per cell was then approximated using equation S5:

$$Cell Metabolic Acitivity= \frac{Fluorescence Intensity}{dsDNA concentration} (S5)$$

The dsDNA concentration was calculated from the PicoGreen® assay as described above.

**4. Water Contact Angle Measurements**

The water contact angle of the scaffolds was tested using the OneAttension Theta optical tensiometer (Biolin Scientific, U.K.). The wettability was tested on three separate hydrogel samples. A 10 μL droplet of deionised water was released onto each hydrogel using an 8-gauge needle (BD Plastics, U.K.). The advancing contact angle was measured over 7.20 seconds.

Figure S7 shows the photograph images of a 10 μL water droplet on the PVPA-*co*-AA hydrogels and the calculated water contact angles are presented in Table S2.

D.

C.

B.

A.

**Figure S7.** Photograph images of 10 μL water droplet on (A) VPA-0, (B) VPA-10, (C) VPA-30 and (D) VPA-50 hydrogels (*n* = 3).

Table S1. Water contact angle of PVPA-*co*-AA hydrogels with increasing VPA content.

| **Sample Code** | **Monomer Feed Ratio (VPA:AA)** | **Water Contact Angle (°)** | | | **Mean ± standard deviation** |
| --- | --- | --- | --- | --- | --- |
|  |  | **1** | **2** | **3** |  |
| VPA-0 | 0:100 | 82.1 | 76.7 | 79.2 | 79.4 ± 2.6 |
| VPA-10 | 10:90 | 69.2 | 66.6 | 67.1 | 67.6 ± 1.4 |
| VPA-30 | 30:70 | 58.2 | 58.3 | 56.4 | 57.7 ± 1.1 |
| VPA-50 | 50:50 | 39.3 | 38.2 | 36.6 | 38.0 ± 1.3 |

**References**

1. J. Serth, M. A. Kuczyk, U. Paeslack, R. Lichtinghagen and U. Jonas, *Am. J. Pathol.*, 2000, **156**, 1189-1196.
